# Socioeconomic and educational influences on malaria prevention and treatment behaviours in rural Nigeria

**DOI:** 10.1186/s12889-025-24326-3

**Published:** 2025-09-24

**Authors:** Jude Oluwapelumi Alao, Oluwadamilare Peter Olowoshile, Timothy Adetayo James, Chinonso Chinaza Okezie, Zainab Pamilerin Adebayo, Success Chimbuchi Ogbonna, Emmanuel Adedeji Oyelayo

**Affiliations:** 1 School of Public Health and Interdisciplinary Studies, Auckland University of Technology, Auckland, New Zealand; 2 Project Enable Africa, Surulere, Lagos State Nigeria; 3 Department of Physics, University of Ilorin, Ilorin, Kwara State Nigeria; 4 Department of Microbiology, Adeleke University, Ede, Osun State Nigeria; 5 Department of Biochemistry, Adeleke University, Ede, Osun State Nigeria; 6 Department of Biochemistry, Ladoke Akintola University of Technology, Ogbomosho, Oyo State Nigeria

**Keywords:** Malaria prevention & control, Insecticide-Treated nets, Socioeconomic factors, Health knowledge, attitudes, practice, Media influence, Health services accessibility

## Abstract

**Background:**

Malaria remains a leading cause of morbidity and mortality in rural Nigeria, where socioeconomic, educational, and informational factors critically influence prevention and treatment behaviours.

**Methods:**

This study is a cross-sectional analysis of the 2021 Malaria Indicator Survey (MIS) to investigate the relationships between household income, education, media exposure, and key malaria control outcomes among rural populations. Three complementary analyses were conducted: household-level associations between income and insecticide-treated net (ITN) ownership and use (*n* = 5,021 households); individual-level associations of education with malaria prevention awareness and ITN use among women aged 15–49 years (*n* = 9,546); and the influence of media exposure on malaria treatment-seeking behaviour (*n* = 893).

**Results:**

Results indicated near-universal ITN ownership across income groups, demonstrating equitable distribution (97.7%), but usage was significantly lower (70.3%). However, ITN use varied inversely with income, with low-income households having 3.6 times higher odds of ITN use (95% CI: 3.07–4.29). Among women, education was a strong predictor of malaria prevention awareness, with higher education levels associated with increased knowledge (p < 0.001). Paradoxically, ITN use decreased as education increased, suggesting that behavioural or contextual factors modulate preventive practice despite greater awareness. Media exposure did not significantly influence the type of healthcare service accessed for malaria treatment, indicating that information access alone may not determine treatment-seeking patterns.

**Conclusions:**

These findings highlight complex relationships between social determinants and malaria control behaviours. While ITN distribution efforts have achieved broad coverage, targeted behavioural interventions may be needed to improve ITN utilisation, particularly among higher-income groups who may underestimate their risk. Education is associated with increased awareness, but this does not necessarily lead to net use, highlighting the need for strategies that address barriers beyond knowledge. The limited impact of media exposure on treatment choices calls for comprehensive approaches that go beyond simply disseminating information, addressing structural and cultural factors in healthcare utilisation. Policymakers, including the Nigerian Ministry of Health (MOH) and the National Malaria Elimination Programme (NMEP), should design interventions that combine education, community engagement, and practical support to ensure sustained behaviour change. This study provides information to help optimise malaria control strategies and reduce the malaria burden in rural Nigeria by addressing both knowledge gaps and real-world barriers to prevention and treatment.

**Supplementary Information:**

The online version contains supplementary material available at 10.1186/s12889-025-24326-3.

## Introduction

Malaria remains a major global public health issue, with an estimated 263 million cases and over 600,000 deaths reported in 2022, predominantly in sub-Saharan Africa [1]. The region accounts for over 90% of global malaria cases and deaths [1]. Within this context, Nigeria bears the highest malaria burden globally, contributing significantly to morbidity and mortality, particularly in rural areas where transmission is intense and healthcare access is limited [2]. Despite ongoing efforts and substantial progress in malaria control, the disease continues to exert a heavy socioeconomic burden, undermining development and perpetuating cycles of poverty [3, 4]. Effective prevention and timely treatment are essential to malaria control strategies to reduce disease incidence and improve health outcomes.

Insecticide-treated nets (ITNs) have been widely recognised as one of the most effective and scalable tools for malaria prevention [5], with mass distribution campaigns significantly increasing access across endemic regions [6]. However, evidence from various settings suggests that ownership of ITNs does not necessarily translate into consistent use, highlighting the connection between behavioural, socioeconomic, and contextual factors in influencing preventive practices [7–10].

Education is an established social determinant of health and has been linked to improved knowledge, attitudes, and practices related to malaria prevention [11, 12]. Higher educational attainment can enhance health literacy and empower individuals to adopt preventive behaviours. Nonetheless, disparities between awareness and actual use of malaria prevention methods have been reported [12], suggesting that education alone may be insufficient to ensure protective practices. Exploring the nuanced relationship between education and malaria prevention behaviours in rural populations remains a priority.

Media exposure constitutes another potentially influential factor in shaping health behaviours by disseminating information and promoting public health messages [13]. Mass media campaigns have been widely used to raise awareness and encourage treatment-seeking for malaria [14]. However, the extent to which media exposure translates into actual healthcare utilisation, particularly in resource-constrained rural settings, is less clear and warrants thorough investigation.

This study utilises nationally representative data from the 2021 Malaria Indicator Survey (MIS) to investigate the relationship between socioeconomic status, education, media exposure, and malaria prevention and treatment behaviours among rural Nigerian populations. Integrating household- and individual-level analyses, we aim to elucidate how these key social determinants influence access to and use of insecticide-treated nets, awareness of prevention strategies, and healthcare utilisation patterns for malaria treatment. This study also seeks to address some unanswered questions in malaria prevention efforts. Why does ITN usage appear to decline with higher income levels, despite near-universal ownership? What cultural or structural barriers may weaken the link between education and preventive health behaviours, such as ITN use or healthcare-seeking? Information gained will inform targeted strategies to enhance malaria control efforts and contribute to Nigeria’s public health goals.

## Methodology

### Data source

This study utilised data from the 2021 MIS, specifically drawing from the Household Recode (HR) and Individual Recode (IR) datasets. These datasets provide comprehensive information on household socioeconomic status, individual education and media exposure, malaria prevention knowledge and behaviours, and treatment-seeking patterns. The combined use of HR and IR datasets enabled a multi-dimensional examination of malaria prevention and treatment behaviours among rural Nigerian populations, focusing on socioeconomic, educational, and informational determinants.

### Study population and sample selection

The analysis was restricted to rural households and individuals residing in rural areas, identified using the place of residence variable (hv025 or v025 = 2). For each analytical component, cases with missing data on key variables were excluded using listwise deletion to ensure complete case analysis and enhance internal validity.

For household-level analyses on malaria prevention (ITN ownership and use), the final sample consisted of 5,021 rural households from the HR dataset. For individual-level analyses of education, awareness, and malaria prevention behaviour, the sample included rural women aged 15–49 years (from the IR dataset), with complete data on education, awareness, and ITN use variables. For analyses exploring the influence of media exposure on malaria treatment-seeking behaviour, the sample comprised 893 rural women who reported having treated malaria and had complete data on media exposure and healthcare access variables.

### Variables

#### Socioeconomic status and malaria prevention (household-level)

The primary independent variable in this component was household income, proxied by the MIS wealth index (hv270). This composite measure is based on asset ownership, housing characteristics, and service access. For analytical clarity, the original five quintiles, poorest, poorer, middle, richer, and richest were recoded into three categories: low income (combining the poorest and poorer quintiles), middle income (the middle quintile), and high income (combining the richer and richest quintiles). This grouping created more precise comparisons between households with similar economic backgrounds. For the exclusion criteria, households with missing ITN ownership or usage data were not included in the analysis. This was important to ensure the data was reliable and that incomplete information would not affect the results.

The dependent variables included two key indicators related to malaria prevention. The first variable was ITN ownership, represented by a binary variable indicating whether the household owned at least one IT, coded as 1 for “Yes” and 0 for “No”. The second dependent variable was ITN use, operationalised as a binary household-level variable created by aggregating individual-level ITN use data from variables HML21$1 through HML21$7. A household was classified as an ITN user if at least one member reported sleeping under an ITN the night before the survey, coded as 1 for Yes and 0 for No.

#### Education and malaria prevention awareness (individual-level)

The primary independent variable was education level, measured by the variable v106, which categorises the highest educational attainment of respondents into four levels: no education, primary, secondary, and higher education.

The dependent variables in this component consisted of malaria prevention awareness and preventive methods. Prevention awareness was defined as a binary variable derived from two indicators: knowledge that malaria can be prevented by sleeping under a mosquito net (ML503A) and knowledge that malaria can be prevented by sleeping under ITN (ML503B). Respondents answering “Yes” to either indicator were classified as aware. The use of prevention was measured by a binary variable indicating whether the respondent slept under any mosquito net (treated or untreated) the night before the survey (ML101), coded as 1 for ‘Yes’ and 0 for ‘No’.

#### Media exposure and malaria treatment-seeking behaviour (individual-level)

Media exposure was operationalised as a composite binary variable based on the reported frequency of engagement with common mass media sources: reading newspapers or magazines (v157), listening to the radio (v158), and watching television (v159). Respondents who reported engaging with any of these media at least once per week were classified as having frequent media exposure, while those with less frequent or no engagement were classified as having no frequent exposure.

The dependent variable in this section was the type of healthcare service accessed for malaria treatment. This was categorised into five groups based on variables H32A1-6 through H32Z1-6: public health services, private clinics or pharmacies, chemists, traditional medicine, and other sources.

### Statistical analysis

All statistical analyses were performed using IBM SPSS Statistics Version 27. Descriptive statistics, including frequencies and percentages, were used to summarise the independent and dependent variables distribution within the rural population samples. Bivariate associations between each independent variable (household income, education, and media exposure) and their respective malaria prevention or treatment outcomes were examined through cross-tabulations. Chi-square tests were applied to assess statistical significance. Row percentages were reported to describe the prevalence of outcomes within each category. Survey weights were applied to account for the complex stratified sampling design used in the MIS datasets. These weights help ensure that the results are representative of the broader population by adjusting for differences in sampling probabilities and non-response.

To estimate independent associations, multivariable analyses were conducted. Binary logistic regression models were fitted to assess the relationships between household income, ITN ownership and use, education, malaria prevention awareness, and net use. Income and education were specified as categorical predictors, with the highest income and lowest education groups serving as reference categories, respectively. A multinomial logistic regression model was employed to analyse media exposure and healthcare access, treating the type of healthcare accessed as the categorical dependent variable and “other sources” as the reference category. Media exposure was entered as a binary predictor representing frequent versus infrequent exposure.

Model diagnostics included reporting adjusted odds ratios (AORs) with 99% confidence intervals (CIs) to quantify the strength and direction of associations. The decision to use 99% CIs, as opposed to the more commonly used 95% CIs, was made to adopt a more conservative approach, reducing the likelihood of Type I errors and ensuring greater robustness in the reported associations. Given the large sample size of the 2021 Malaria Indicator Survey (MIS), where even small effects could become statistically significant, the use of 99% CIs helps emphasize more meaningful and substantial associations. Model fit was evaluated using Nagelkerke R² for binary logistic regression models and pseudo R² statistics for the multinomial model. The Hosmer-Lemeshow goodness-of-fit test was applied to binary logistic regression models. Multicollinearity diagnostics were performed to ensure that model assumptions were met and to identify any violations that could affect the robustness of the results.

Data visualisations were created using Python with matplotlib in Jupyter Notebook version 7.0.8. Categorical data were displayed primarily as grouped bar charts to compare subcategories within income and education groups. Percentage values were annotated on bars for clarity, and clear labels and titles were included to ensure interpretability.

## Results

### Socioeconomic status and malaria prevention among rural households

The analysis included 5,021 rural households from 2021 Nigerian MIS after excluding cases with missing data on household income, ITN ownership, or ITN use (Supplementary Material 1). Nearly half of the households (47.6%) were classified as low-income, 22.8% as middle-income, and 29.6% as high-income, reflecting substantial economic diversity within rural communities (Table 1). ITN ownership was nearly universal, with 97.7% of households reporting possessing at least one ITN. However, actual ITN use was lower, with 83.0% of households indicating that at least one member had slept under an ITN the previous night.

**Table 1:** Distribution of household income, ITN ownership, and ITN use

Variable	Category	Frequency (*n*)	Percentage (%)
Household Income	Low Income	2,391	47.6
	Middle Income	1,143	22.8
	High Income	1,487	29.6
	Total	5,021	100.0
ITN Ownership	Owns at least one ITN	4,905	97.7
	Does not own ITN	116	2.3
	Total	5,021	100.0
ITN Use	Used ITN last night	4,168	83.0
	Did not use ITN	853	17.0
	**Total**	**5**,**021**	**100.0**

Bivariate analysis revealed that ITN ownership was consistently high across all income groups: 98.0% in low-income households, 97.5% in middle-income households, and 97.3% in high-income households, with no statistically significant differences observed (χ² (2) = 2.468, *p* > 0.001). This indicates equitable distribution of ITNs regardless of household income.

**Fig. 1:**
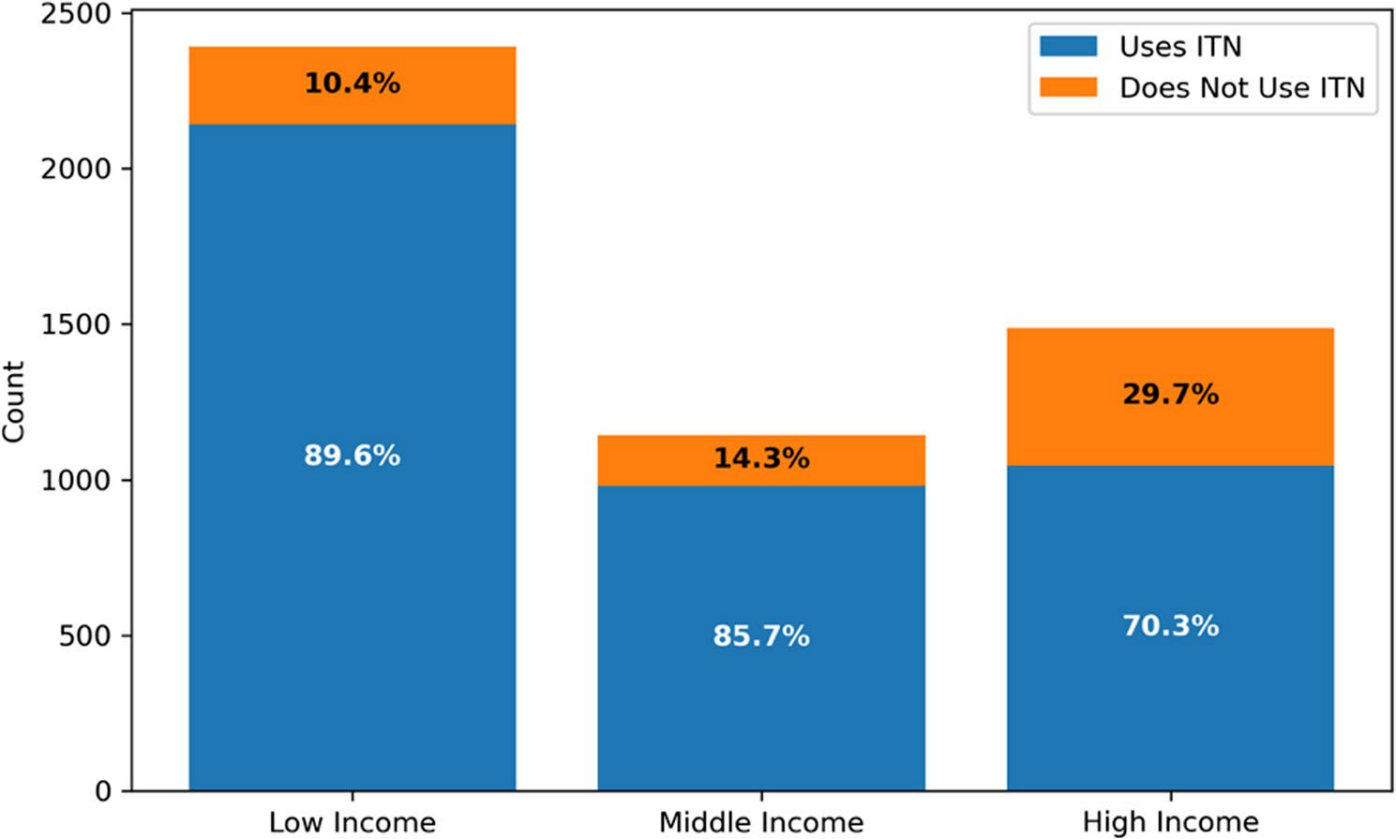
Proportion of ITN usage across income levels

In contrast, ITN use varied significantly by income category (χ² (2) = 248.539, *p* < 0.001), with the highest usage in low-income households (89.6%), followed by middle-income households (85.7%), and the lowest in high-income households (70.3%) (Fig. 1). Despite universal access, this inverse relationship between income and ITN use highlights a behavioural disparity.

Multivariable logistic regression confirmed these trends. Household income was not a significant predictor of ITN ownership, with an AOR for low- and middle-income households compared to high-income households being 1.379 (95% CI: 0.90–2.12, *p* = 0.140) and 1.062 (99% CI: 0.66–1.72, *p* > 0.001), respectively. However, income strongly predicted ITN use, with low-income households having over threefold higher odds of ITN use than high-income households (AOR = 3.627, 95% CI: 3.07–4.29, *p* < 0.001), and middle-income households showing more than double the odds (AOR = 2.535, 95% CI: 2.06–3.13, *p* < 0.001).

### Education and malaria prevention awareness and behaviour among rural women

**Fig. 2:**
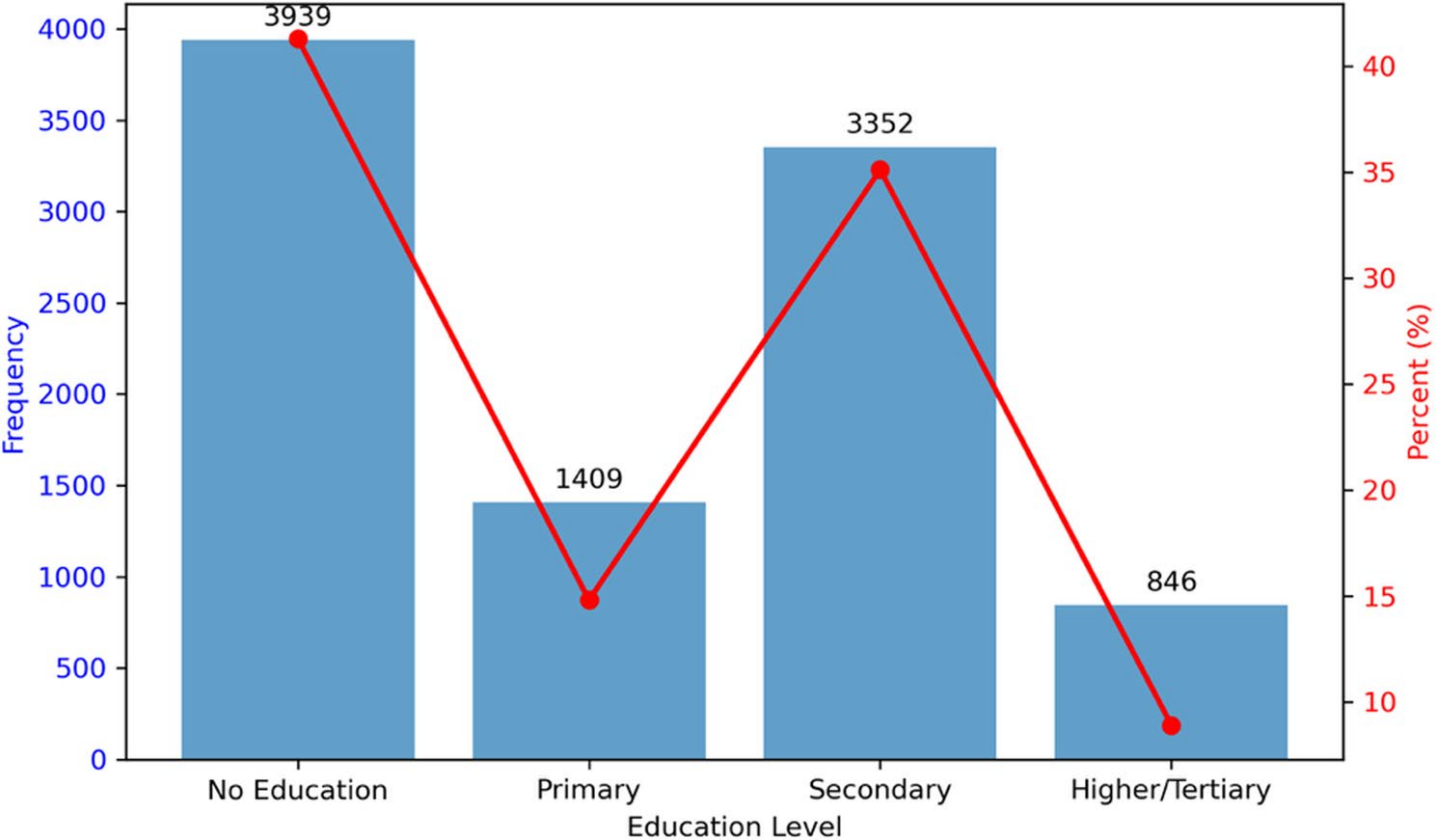
Comparison of education levels by count and percentage

A total of 9,546 rural women aged 15–49 years were analysed for education-related malaria prevention outcomes (Supplementary Material 2). The largest group had no formal education (41.3%), followed by secondary (35.1%), primary (14.8%), and tertiary education (8.9%) (Fig. 2).

**Fig. 3:**
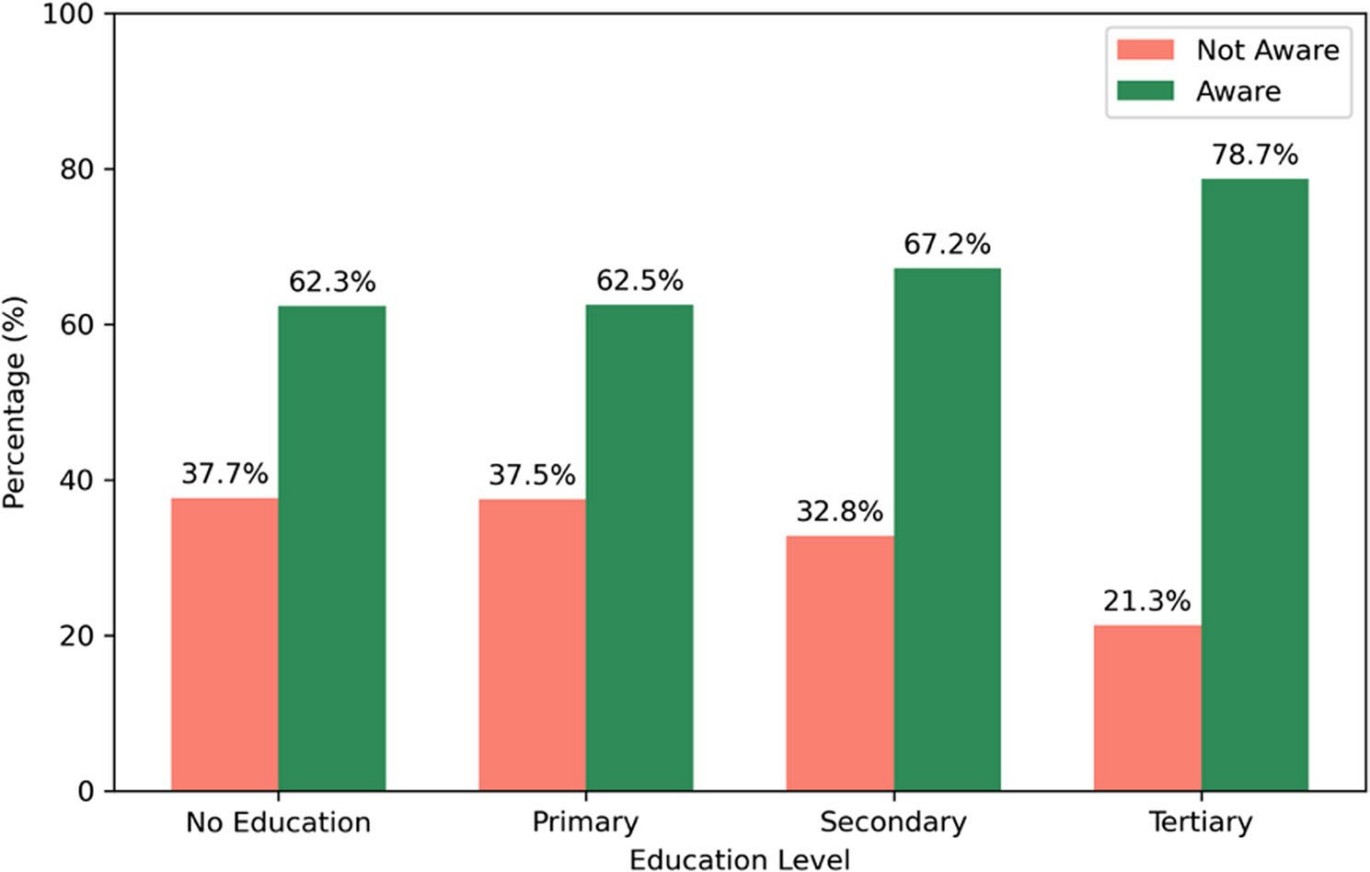
Relationship between education and malaria prevention awareness

In total, 65.5% of women were aware that malaria prevention includes sleeping under a mosquito net or ITN, with awareness increasing significantly by education level (χ² = 93.451, *p* < 0.001). Awareness ranged from 62.3% among women without education to 78.7% in those with tertiary education (Fig. 3).

**Fig. 4:**
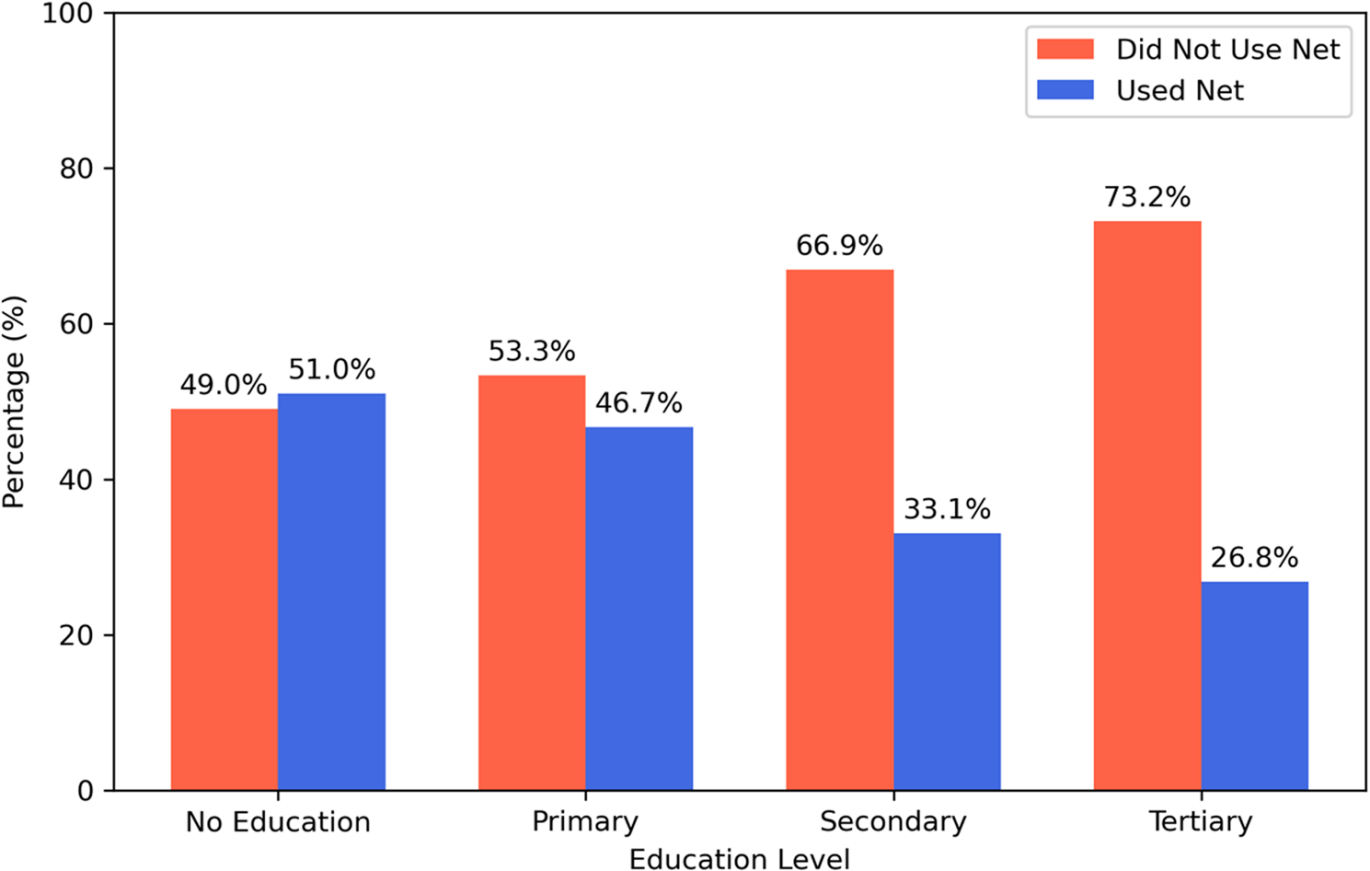
Relationship between education level and ITN use

Contrary to awareness patterns, reported use of mosquito nets the previous night decreased with higher education levels (χ² = 331.761, *p* < 0.001). Usage was highest among women with no education (51.0%) and lowest in those with tertiary education (26.8%) (Fig. 4).

Adjusted logistic regression models controlling for age, wealth, and region demonstrated that education strongly predicted malaria prevention awareness. Women with primary, secondary, and tertiary education had significantly lower odds of being unaware compared to those with no education (inverted AORs of 0.447, 0.450, and 0.554, respectively; all *p* < 0.001) (Table 2).

**Table 2:** Effect of education level on ITN use

Education Level	AOR	95% CI	*p*-value
Primary vs. None	0.447	[0.375–0.535]	< 0.001
Secondary vs. None	0.450	[0.368–0.550]	< 0.001
Tertiary vs. None	0.554	[0.434–0.707]	< 0.001

For ITN use, education remained a significant predictor, but with attenuated odds. Women with primary and secondary education had higher odds of net use than those with no education (AOR = 2.836 and 2.389, respectively), while tertiary education showed a smaller but still significant effect (AOR = 1.350) (Table 3).

**Table 3:** Logistic regression model – ITN use

Education Level	AOR	99% CI	*p*-value
Primary vs. None	2.836	[2.38–3.38]	< 0.001
Secondary vs. None	2.389	[1.95–2.93]	< 0.001
Tertiary vs. None	1.350	[1.15–1.59]	< 0.001

### Media exposure and health-seeking behaviour for malaria treatment

The final sample for this component consisted of 893 rural respondents with complete data on media exposure and access to malaria treatment (Supplementary Material 3). Most respondents (63.9%) reported no frequent media exposure, while 36.1% reported frequent exposure to at least one form of media such as radio, television, or newspapers (Table 4). Regarding health-seeking behaviour, public health services were the most utilised for malaria treatment (49.3%), followed by private clinics/pharmacies (26.8%), chemists (19.9%), traditional medicine (2.5%), and other sources (1.6%).

**Table 4:** Frequency distribution of media exposure and health access

Variable	Category	Frequency	Percent (%)
Media Exposure	No Frequent Media Exposure	571	63.9
	Frequent Media Exposure	322	36.1
Health Access	Public Health Services	440	49.3
	Private Hospitals/Pharmacies/Private Services	239	26.8
	Chemists	178	19.9
	Traditional Medicine	22	2.5
	Other Sources	14	1.6
	**Total**	**893**	**100.0**

Bivariate analysis revealed no statistically significant differences in the type of health service utilised between those with and without frequent media exposure (χ² (4) = 0.508, *p* > 0.001) (Table 5). Individuals without frequent media exposure comprised most users across all service categories.

**Table 5:** Cross-tabulation of media exposure by type of health service used

Health Service Type	No Frequent Media Exposure (%)	Frequent Media Exposure (%)	Total (*N*)
Public Health Services	64.5	35.5	440
Private Clinics/Pharmacies/Private Services	62.3	37.7	239
Chemists	64.0	36.0	178
Traditional Medicine	68.2	31.8	22
Other Sources	64.3	35.7	14
Total	**63.9**	**36.1**	**893**

Multinomial logistic regression further confirmed that media exposure did not significantly predict the type of healthcare service accessed. Adjusted odds ratios for frequent media exposure relative to no exposure were non-significant across public health services (AOR = 1.011, *p* > 0.001), private clinics (AOR = 0.920, *p* > 0.001), chemists (AOR = 0.990, *p* > 0.001), and traditional medicine (AOR = 1.190, *p* > 0.001). The model had negligible explanatory power (Nagelkerke R² = 0.001).

## Discussion

The findings of this study reveal important nuances in access to and use of ITNs, malaria prevention awareness, and treatment-seeking behaviours, highlighting gaps and inequities that can inform public health policy and intervention strategies. Our analysis demonstrated near-universal ITN ownership across rural households, irrespective of income level, confirming the success of Nigeria’s mass ITN distribution campaigns and their equitable reach in rural areas. This finding aligns with other studies reporting widespread ITN access following intensified national malaria control efforts [15, 16]. The absence of a significant association between income and ITN ownership suggests that physical availability and distribution channels effectively minimise economic barriers to access.

Despite this equitable ownership, a significant and inverse association was observed between household income and ITN use, with lower-income households substantially more likely to report using ITNs than higher-income households. This counterintuitive finding contrasts with common assumptions that higher socioeconomic status correlates positively with preventive health behaviours [17, 18]. Several plausible explanations exist. Lower-income households may perceive a higher risk or burden of malaria due to poorer housing conditions or limited alternative protective measures, motivating consistent ITN use [19]. Conversely, higher-income households may have access to alternative preventive measures, such as window screens, air conditioning, or repellents, or may underestimate their personal malaria risk, leading to lower ITN utilisation. Behavioural factors, including discomfort or inconvenience associated with net use in warmer weather, may also disproportionately affect wealthier households with different housing designs or lifestyle patterns. The findings underscore the importance of complementing ITN distribution with targeted behavioural interventions, particularly in higher-income rural populations, to address misconceptions, promote consistent use, and emphasise universal protection benefits.

Among rural women, educational attainment was strongly associated with malaria prevention awareness, confirming education as a vital determinant of health knowledge. Women with primary, secondary, and tertiary education had significantly higher odds of being aware of malaria prevention methods compared to those with no education. This finding is consistent with extensive literature that links education to improved health literacy and the capacity to understand disease transmission and prevention [20, 21].

However, the inverse relationship between education and reported net use contrasts with the positive association between education and awareness. Women with no education were more likely to sleep under a mosquito net the night before the survey than those with higher education levels. This discrepancy may reflect complex sociocultural and contextual factors. For example, women with higher education may live in households with better housing and reduced mosquito exposure, lowering their perceived need for nets. Alternatively, they might engage in occupational or social activities that reduce net use during peak mosquito hours or perceive nets as inconvenient. Seasonal fluctuations, reported discomfort, or alternative preventive measures may also contribute. These findings show that increased knowledge does not automatically translate to preventive behaviour, emphasising the need for interventions that bridge the gap between awareness and consistent practice.

While media exposure is often assumed to be a key driver of health behaviour change [22], this study found no significant association between frequency of media exposure and the type of healthcare service accessed for malaria treatment among rural residents. This lack of association persisted after adjusting for potential confounders in multinomial logistic regression models. This finding may indicate that while media campaigns effectively disseminate knowledge, structural factors such as healthcare availability, cost, cultural beliefs, and trust in providers may play more decisive roles in healthcare utilisation choices [23–25]. It also suggests that in rural Nigerian contexts, information accessed through media may be insufficient to overcome barriers related to accessibility and acceptability of services. These results align with studies reporting limited translation of health communication into behavioural change without addressing systemic obstacles [26, 27].

The study’s findings have several practical implications. First, maintaining equitable ITN distribution remains essential; however behavioural strategies must target socioeconomic groups exhibiting lower ITN use, particularly wealthier households, to achieve sustained malaria control. Tailored messaging that addresses perceptions, convenience, and alternative protection strategies may enhance ITN adherence. Second, increasing malaria prevention awareness through education is critical, but insufficient alone to ensure net use. Comprehensive community-based programs are needed to integrate knowledge with behaviour change communication, social norm interventions, and address structural constraints. Third, enhancing access to and quality healthcare services remains vital, but interventions should also recognise that mass media exposure alone may not significantly shift treatment-seeking behaviours.

There are several reasons why media exposure was not significantly associated with treatment-seeking behaviour in this study. One possibility is that the media messages may not have been culturally or linguistically relevant to the target population, leading to a disconnect between the message and the community’s needs. Even when media campaigns are widespread, the content may not resonate with local values, beliefs, or language, limiting its effectiveness. Another reason could be the structural barriers to healthcare access. Even if individuals are aware of the importance of seeking treatment, limited access to healthcare facilities, due to distance, cost, or insufficient healthcare infrastructure, can prevent them from acting on that knowledge. In many rural areas, these barriers can be just as significant as awareness, if not more so. Lastly, passive exposure to media without active engagement or trust in the messaging may also contribute to the lack of significant impact. If individuals do not engage with the media content or are sceptical of its source, the effect on their treatment-seeking behaviours will be minimal. This underscores the importance of increasing media exposure and ensuring that messages are engaging, credible, and trusted by the target audience.

This study has limitations. The cross-sectional design precludes causal inference, and reliance on self-reported measures may introduce recall and social desirability biases, particularly in ITN use and treatment-seeking behaviours. The wealth index, while a standard proxy for socioeconomic status, may not capture all dimensions of economic variation. Furthermore, media exposure was broadly defined and did not assess content, message quality, or comprehension.

Additionally, several unmeasured confounders may influence the observed relationships. For example, differences in local malaria transmission intensity could affect both ITN use and treatment-seeking behaviour. Areas with higher transmission rates may see greater motivation for consistent ITN use and more frequent treatment-seeking, regardless of socioeconomic factors. Seasonal variability in malaria incidence may also impact behaviours, with higher transmission during certain seasons driving more active efforts to prevent and treat malaria. Household size and composition might also play a role, as larger households may face different resource constraints or have varying levels of access to malaria prevention tools. Clustering effects, such as the influence of local community health practices or proximity to healthcare services, could also introduce bias by grouping individuals with similar behaviours or exposures, affecting the generalisability of findings.

In addition, the media exposure analysis, with a sample size of *n* = 893, is smaller than the other analyses in this study. As such, its findings should be interpreted with caution, and the conclusions drawn from this smaller sample may not be as generalizable. Further research with larger sample sizes is needed to confirm these findings. While we account for the most significant measured factors, these unmeasured variables could limit the strength of the conclusions and suggest the need for further studies to explore these influences.

Future research should employ longitudinal designs to capture temporal changes in malaria prevention behaviours more effectively. This would help identify trends over time and allow for a better understanding of how factors such as income, education, and media exposure influence ITN use and treatment-seeking decisions. In addition, qualitative studies exploring the behavioural drivers and barriers across different socioeconomic groups could offer valuable insights into the reasons behind the differences in ITN use and healthcare choices.

Community-driven campaigns and interventions that are tailored to the local culture could be especially useful in improving the use of ITNs, particularly in higher-income or more educated households where awareness of malaria does not always lead to action. These campaigns should focus on addressing the unique challenges faced by these groups, such as the belief that they are less at risk of malaria or the practical difficulties in using ITNs. The Nigerian Ministry of Health (MOH) and the National Malaria Elimination Programme (NMEP) can play a key role in targeting these groups to boost ITN usage across all income levels. In addition, evaluating interventions that combine education, behaviour change, and improving healthcare access, along with stronger community involvement, can help fine-tune malaria control efforts. Understanding and addressing both the knowledge gaps and real-world barriers to using ITNs will enable these programs to make a lasting difference. With the right strategies, the MOH and NMEP can enhance the effectiveness of malaria prevention programs, creating approaches that resonate with local communities and could be applied to other areas facing similar challenges.

## Conclusion

This study highlights significant gaps in the relationship between ITN distribution and actual use in rural Nigeria, particularly among higher-income groups. Although education is crucial in increasing awareness about malaria prevention, it does not consistently lead to protective behaviours such as ITN use. Additionally, the lack of significant impact from media exposure on treatment-seeking behaviour points to the need for more nuanced strategies beyond informational campaigns.

To improve malaria control efforts, policymakers and program implementers, including MOH and NMEP, should design targeted behavioural interventions that address both perceived and practical barriers to ITN use across all socioeconomic groups. These interventions should focus particularly on wealthier households that may underestimate their risk of malaria and are less likely to use ITNs consistently. Educational programs should not only provide information but also integrate community engagement, practical support, and culturally relevant messaging to encourage sustained behaviour change. Additionally, improving healthcare access and building trust in formal healthcare services requires comprehensive strategies that go beyond mass media exposure. Structural barriers, such as healthcare accessibility, affordability, and cultural factors, must be addressed to ensure that increased awareness translates into real action. Focusing on these factors allows the MOH and NMEP to develop effective, culturally tailored programs that resonate with local communities, making malaria prevention more accessible and sustainable for all.

Future research should consider longitudinal or mixed-methods studies to better capture the dynamics of malaria prevention and treatment-seeking behaviours over time. Exploring the underlying drivers of these behaviours through qualitative research could provide valuable insights into the barriers and facilitators of effective malaria control. By tackling these complex social determinants, it is possible to achieve lasting reductions in malaria incidence and promote health equity in rural Nigeria.

## Supplementary Information

Supplementary Material 1.

Supplementary Material 2.

Supplementary Material 3.

## Data Availability

Data is provided within the manuscript and supplementary information files.
