# Supplementary material for: Socioeconomic and educational influences on malaria prevention and treatment behaviours in rural Nigeria
Source: BMC Public Health. 2025 Sep 24;25:3079. doi: 10.1186/s12889-025-24326-3 (PMC12462100; doi:10.1186/s12889-025-24326-3)
Supplement: Supplementary file 1 — Supplementary Material 1. [file 12889_2025_24326_MOESM1_ESM.docx]

USE ALL.

COMPUTE filter_$=(hv025 = 2 AND NOT MISSING(wealth_3cat)AND NOT MISSING(own_itn)AND NOT MISSING(net_use)).

>Error # 4285 in column 45. Text: wealth_3cat

>Incorrect variable name: either the name is more than 64 characters, or it is

>not defined by a previous command.

>Execution of this command stops.

VARIABLE LABELS filter_$ 'hv025 = 2 AND NOT MISSING(wealth_3cat)AND NOT MISSING(own_itn)A ND NOT '+

'MISSING(net_use) (FILTER)'.

VALUE LABELS filter_$ 0 'Not Selected' 1 'Selected'. FORMATS filter_$ (f1.0).

FILTER BY filter_$. EXECUTE.

FREQUENCIES VARIABLES=INCOME ITN_ownership ITN_use

/ORDER=ANALYSIS.

**Frequencies**

# Statistics

| INCOME | | | ITN_ownership | ITN_use |
| --- | --- | --- | --- | --- |
| N | Valid | 5021 | 5021 | 5021 |
|  | Missing | 0 | 0 | 0 |

**Frequency Table**

**INCOME**

| Frequency | | | Percent | Valid Percent | Cumulative Percent |
| --- | --- | --- | --- | --- | --- |
| Valid | Low Income | 2391 | 47.6 | 47.6 | 47.6 |
|  | Middle Income | 1143 | 22.8 | 22.8 | 70.4 |
|  | High Income | 1487 | 29.6 | 29.6 | 100.0 |
|  | Total | 5021 | 100.0 | 100.0 |  |

**ITN_ownership**

| Frequency | | | Percent | Valid Percent | Cumulative Percent |
| --- | --- | --- | --- | --- | --- |
| Valid | Does not own ITN | 116 | 2.3 | 2.3 | 2.3 |
|  | Owns ITN | 4905 | 97.7 | 97.7 | 100.0 |
|  | Total | 5021 | 100.0 | 100.0 |  |

**ITN_use**

| Frequency | | | Percent | Valid Percent | Cumulative Percent |
| --- | --- | --- | --- | --- | --- |
| Valid | Does not use ITN | 853 | 17.0 | 17.0 | 17.0 |
|  | Uses ITN | 4168 | 83.0 | 83.0 | 100.0 |
|  | Total | 5021 | 100.0 | 100.0 |  |

CROSSTABS

/TABLES=INCOME BY ITN_ownership

/FORMAT=AVALUE TABLES

/STATISTICS=CHISQ

/CELLS=COUNT ROW

/COUNT ROUND CELL.

**Crosstabs**

# Case Processing Summary

Cases

| Valid | | | Missing | | Total | |
| --- | --- | --- | --- | --- | --- | --- |
| N | | Percent | N | Percent | N | Percent |
| INCOME * ITN_ownership | 5021 | 100.0% | 0 | 0.0% | 5021 | 100.0% |

# INCOME * ITN_ownership Crosstabulation

| ITN_ownership | | | | | Total |
| --- | --- | --- | --- | --- | --- |
| Does not own ITN | | | | Owns ITN |  |
| INCOME | Low Income | Count | 47 | 2344 | 2391 |
|  |  | % within INCOME | 2.0% | 98.0% | 100.0% |
|  | Middle Income | Count | 29 | 1114 | 1143 |
|  |  | % within INCOME | 2.5% | 97.5% | 100.0% |
|  | High Income | Count | 40 | 1447 | 1487 |
|  |  | % within INCOME | 2.7% | 97.3% | 100.0% |
| Total | | Count | 116 | 4905 | 5021 |
|  |  | % within INCOME | 2.3% | 97.7% | 100.0% |

**Chi-Square Tests**

| Value | | df | Asymptotic Significance (2- sided) |
| --- | --- | --- | --- |
| Pearson Chi-Square | 2.468a | 2 | .291 |
| Likelihood Ratio | 2.480 | 2 | .289 |
| Linear-by-Linear Association | 2.299 | 1 | .129 |
| N of Valid Cases | 5021 |  |  |

a. 0 cells (.0%) have expected count less than 5. The minimum expected count is 26.41.

CROSSTABS

/TABLES=INCOME BY ITN_ownership

/FORMAT=AVALUE TABLES

/STATISTICS=CHISQ

/CELLS=COUNT ROW

/COUNT ROUND CELL

/BARCHART.

**Crosstabs**

# Case Processing Summary

Cases

| Valid | | | Missing | | Total | |
| --- | --- | --- | --- | --- | --- | --- |
| N | | Percent | N | Percent | N | Percent |
| INCOME * ITN_ownership | 5021 | 100.0% | 0 | 0.0% | 5021 | 100.0% |

# INCOME * ITN_ownership Crosstabulation

| ITN_ownership | | | | | Total |
| --- | --- | --- | --- | --- | --- |
| Does not own ITN | | | | Owns ITN |  |
| INCOME | Low Income | Count | 47 | 2344 | 2391 |
|  |  | % within INCOME | 2.0% | 98.0% | 100.0% |
|  | Middle Income | Count | 29 | 1114 | 1143 |
|  |  | % within INCOME | 2.5% | 97.5% | 100.0% |
|  | High Income | Count | 40 | 1447 | 1487 |
|  |  | % within INCOME | 2.7% | 97.3% | 100.0% |
| Total | | Count | 116 | 4905 | 5021 |
|  |  | % within INCOME | 2.3% | 97.7% | 100.0% |

**Chi-Square Tests**

| Value | | df | Asymptotic Significance (2- sided) |
| --- | --- | --- | --- |
| Pearson Chi-Square | 2.468a | 2 | .291 |
| Likelihood Ratio | 2.480 | 2 | .289 |
| Linear-by-Linear Association | 2.299 | 1 | .129 |
| N of Valid Cases | 5021 |  |  |

a. 0 cells (.0%) have expected count less than 5. The minimum expected count is 26.41.

2,500

2,000

1,500

**Count**

1,000

500

**Bar Chart**

0

**ITN_ownership**

Does not own ITN Owns ITN

Low Income

Middle Income

**INCOME**

High Income

CROSSTABS

/TABLES=INCOME BY ITN_use

/FORMAT=AVALUE TABLES

/STATISTICS=CHISQ

/CELLS=COUNT ROW

/COUNT ROUND CELL

/BARCHART.

**Crosstabs**

# Case Processing Summary

Cases

| Valid | | | Missing | | Total | |
| --- | --- | --- | --- | --- | --- | --- |
| N | | Percent | N | Percent | N | Percent |
| INCOME * ITN_use | 5021 | 100.0% | 0 | 0.0% | 5021 | 100.0% |

# INCOME * ITN_use Crosstabulation

| ITN_use | | | | | Total |
| --- | --- | --- | --- | --- | --- |
| Does not use ITN | | | | Uses ITN |  |
| INCOME | Low Income | Count | 249 | 2142 | 2391 |
|  |  | % within INCOME | 10.4% | 89.6% | 100.0% |
|  | Middle Income | Count | 163 | 980 | 1143 |
|  |  | % within INCOME | 14.3% | 85.7% | 100.0% |
|  | High Income | Count | 441 | 1046 | 1487 |
|  |  | % within INCOME | 29.7% | 70.3% | 100.0% |
| Total | | Count | 853 | 4168 | 5021 |
|  |  | % within INCOME | 17.0% | 83.0% | 100.0% |

**Chi-Square Tests**

| Value | | df | Asymptotic Significance (2- sided) |
| --- | --- | --- | --- |
| Pearson Chi-Square | 248.539a | 2 | .000 |
| Likelihood Ratio | 234.113 | 2 | .000 |
| Linear-by-Linear Association | 227.888 | 1 | .000 |
| N of Valid Cases | 5021 |  |  |

a. 0 cells (.0%) have expected count less than 5. The minimum expected count is 194.18.

2,500

2,000

1,500

**Count**

1,000

500

**Bar Chart**

0

|  | | | | | | | **ITN_use**  Does not use ITN Uses ITN |
| --- | --- | --- | --- | --- | --- | --- | --- |
|  | | | | | | |  |
|  |  |  | | | | |  |
|  |  |  | | | | |  |
|  |  |  | | | | |  |
|  |  |  |  |  |  |  |  |
|  |  |  |  |  |  |  |  |

Low Income

Middle Income

**INCOME**

High Income

LOGISTIC REGRESSION VARIABLES ITN_ownership

/METHOD=ENTER INCOME

/CONTRAST (INCOME)=Indicator

/CRITERIA=PIN(.05) POUT(.10) ITERATE(20) CUT(.5).

**Logistic Regression**

# Case Processing Summary

| Unweighted Casesa N | | | Percent |
| --- | --- | --- | --- |
| Selected Cases | Included in Analysis | 5021 | 100.0 |
|  | Missing Cases | 0 | .0 |
|  | Total | 5021 | 100.0 |
| Unselected Cases | | 0 | .0 |
| Total | | 5021 | 100.0 |

1. If weight is in effect, see classification table for the total number of cases.

# Dependent Variable Encoding

Original Value Internal Value

| Does not own ITN | 0 |
| --- | --- |
| Owns ITN | 1 |

# Categorical Variables Codings

| Frequency | | | Parameter coding | |
| --- | --- | --- | --- | --- |
|  |  |  | (1) | (2) |
| INCOME | Low Income | 2391 | 1.000 | .000 |
|  | Middle Income | 1143 | .000 | 1.000 |
|  | High Income | 1487 | .000 | .000 |

**Block 0: Beginning Block**

# Classification Tablea,b

Predicted

| ITN_ownership | | | |  |
| --- | --- | --- | --- | --- |
| Observed |  | Does not own ITN | Owns ITN | Percentage Correct |
| Step 0 ITN_ownership | Does not own ITN | 0 | 116 | .0 |
|  | Owns ITN | 0 | 4905 | 100.0 |
| Overall Percentage |  |  |  | 97.7 |

1. Constant is included in the model.
2. The cut value is .500

# Variables in the Equation

| B | | S.E. | Wald | df | Sig. | Exp(B) |
| --- | --- | --- | --- | --- | --- | --- |
| Step 0 Constant | 3.744 | .094 | 1588.825 | 1 | .000 | 42.284 |

**Variables not in the Equation**

| Score | | | | df | Sig. |
| --- | --- | --- | --- | --- | --- |
| Step 0 | Variables | INCOME | 2.468 | 2 | .291 |
|  |  | INCOME(1) | 2.402 | 1 | .121 |
|  |  | INCOME(2) | .338 | 1 | .561 |
|  | Overall Statistics | | 2.468 | 2 | .291 |

**Block 1: Method = Enter**

# Omnibus Tests of Model Coefficients

| Chi-square | | | df | Sig. |
| --- | --- | --- | --- | --- |
| Step 1 | Step | 2.480 | 2 | .289 |
|  | Block | 2.480 | 2 | .289 |
|  | Model | 2.480 | 2 | .289 |

**Model Summary**

| -2 Log  Step likelihood | | Cox & Snell R Square | Nagelkerke R Square |
| --- | --- | --- | --- |
| 1 | 1100.948a | .000 | .003 |

1. Estimation terminated at iteration number 7 because parameter estimates changed by less than .001.

# Classification Tablea

Predicted

| ITN_ownership | | | |  |
| --- | --- | --- | --- | --- |
| Observed |  | Does not own ITN | Owns ITN | Percentage Correct |
| Step 1 ITN_ownership | Does not own ITN | 0 | 116 | .0 |
|  | Owns ITN | 0 | 4905 | 100.0 |
| Overall Percentage |  |  |  | 97.7 |

1. The cut value is .500

# Variables in the Equation

| B | | | S.E. | Wald | df | Sig. | Exp(B) |
| --- | --- | --- | --- | --- | --- | --- | --- |
| Step 1a | INCOME |  |  | 2.451 | 2 | .294 |  |
|  | INCOME(1) | .321 | .218 | 2.175 | 1 | .140 | 1.379 |
|  | INCOME(2) | .060 | .247 | .059 | 1 | .808 | 1.062 |
|  | Constant | 3.588 | .160 | 501.201 | 1 | .000 | 36.175 |

1. Variable(s) entered on step 1: INCOME.

LOGISTIC REGRESSION VARIABLES ITN_use

/METHOD=ENTER INCOME

/CONTRAST (INCOME)=Indicator

/CRITERIA=PIN(.05) POUT(.10) ITERATE(20) CUT(.5).

**Logistic Regression**

# Case Processing Summary

| Unweighted Casesa N | | | Percent |
| --- | --- | --- | --- |
| Selected Cases | Included in Analysis | 5021 | 100.0 |
|  | Missing Cases | 0 | .0 |
|  | Total | 5021 | 100.0 |
| Unselected Cases | | 0 | .0 |
| Total | | 5021 | 100.0 |

1. If weight is in effect, see classification table for the total number of cases.

# Dependent Variable Encoding

Original Value Internal Value

| Does not use ITN | 0 |
| --- | --- |
| Uses ITN | 1 |

# Categorical Variables Codings

| Frequency | | | Parameter coding | |
| --- | --- | --- | --- | --- |
|  |  |  | (1) | (2) |
| INCOME | Low Income | 2391 | 1.000 | .000 |
|  | Middle Income | 1143 | .000 | 1.000 |
|  | High Income | 1487 | .000 | .000 |

**Block 0: Beginning Block**

# Classification Tablea,b

Predicted

ITN_use

Does not use

ITN Uses ITN

Percentage Correct

Observed

Step 0 ITN_use Does not use ITN

Uses ITN

Overall Percentage

83.0

100.0

4168

0

.0

853

0

1. Constant is included in the model.
2. The cut value is .500

# Variables in the Equation

| B | | S.E. | Wald | df | Sig. | Exp(B) |
| --- | --- | --- | --- | --- | --- | --- |
| Step 0 Constant | 1.586 | .038 | 1782.089 | 1 | .000 | 4.886 |

**Variables not in the Equation**

| Score | | | | df | Sig. |
| --- | --- | --- | --- | --- | --- |
| Step 0 | Variables | INCOME | 248.539 | 2 | .000 |
|  |  | INCOME(1) | 139.912 | 1 | .000 |
|  |  | INCOME(2) | 7.809 | 1 | .005 |
|  | Overall Statistics | | 248.539 | 2 | .000 |

**Block 1: Method = Enter**

# Omnibus Tests of Model Coefficients

| Chi-square | | | df | Sig. |
| --- | --- | --- | --- | --- |
| Step 1 | Step | 234.113 | 2 | .000 |
|  | Block | 234.113 | 2 | .000 |
|  | Model | 234.113 | 2 | .000 |

**Model Summary**

| -2 Log  Step likelihood | | Cox & Snell R Square | Nagelkerke R Square |
| --- | --- | --- | --- |
| 1 | 4342.088a | .046 | .076 |

1. Estimation terminated at iteration number 5 because parameter estimates changed by less than .001.

# Classification Tablea

Predicted

ITN_use

Does not use

ITN Uses ITN

Percentage Correct

Observed

Step 1 ITN_use Does not use ITN

Uses ITN

Overall Percentage

83.0

100.0

4168

0

.0

853

0

1. The cut value is .500

# Variables in the Equation

| B | | | S.E. | Wald | df | Sig. | Exp(B) |
| --- | --- | --- | --- | --- | --- | --- | --- |
| Step 1a | INCOME |  |  | 232.332 | 2 | .000 |  |
|  | INCOME(1) | 1.288 | .088 | 215.385 | 1 | .000 | 3.627 |
|  | INCOME(2) | .930 | .102 | 83.353 | 1 | .000 | 2.535 |
|  | Constant | .864 | .057 | 231.403 | 1 | .000 | 2.372 |

1. Variable(s) entered on step 1: INCOME.
