# Supplementary material for: Socioeconomic and educational influences on malaria prevention and treatment behaviours in rural Nigeria
Source: BMC Public Health. 2025 Sep 24;25:3079. doi: 10.1186/s12889-025-24326-3 (PMC12462100; doi:10.1186/s12889-025-24326-3)
Supplement: Supplementary file 2 — Supplementary Material 2. [file 12889_2025_24326_MOESM2_ESM.docx]

USE ALL.

COMPUTE filter_$=(v025 = 2 AND NOT MISSING(media_exposure) AND NOT MISSING (health_access

) ).

VARIABLE LABELS filter_$ 'v025 = 2 AND NOT MISSING(media_exposure) AND NOT MISSING '+ '(health_access) (FILTER)'.

VALUE LABELS filter_$ 0 'Not Selected' 1 'Selected'. FORMATS filter_$ (f1.0).

FILTER BY filter_$. EXECUTE.

FREQUENCIES VARIABLES=media_exposure Health_service

/ORDER=ANALYSIS.

**Frequencies**

# Statistics

| media_exposur e | | | Health_service |
| --- | --- | --- | --- |
| N | Valid | 893 | 893 |
|  | Missing | 0 | 0 |

**Frequency Table**

# media_exposure

| Frequency | | | Percent | Valid Percent | Cumulative Percent |
| --- | --- | --- | --- | --- | --- |
| Valid | No Frequent Media Exposure | 571 | 63.9 | 63.9 | 63.9 |
|  | Frequent Media Exposure | 322 | 36.1 | 36.1 | 100.0 |
|  | Total | 893 | 100.0 | 100.0 |  |

**Health_service**

| Frequency | | | Percent | Valid Percent | Cumulative Percent |
| --- | --- | --- | --- | --- | --- |
| Valid | Public Health Services | 440 | 49.3 | 49.3 | 49.3 |
|  | Private Clinics\Pharmacies\Private Health Services | 239 | 26.8 | 26.8 | 76.0 |
|  | Chemists | 178 | 19.9 | 19.9 | 96.0 |
|  | Traditional Medicine | 22 | 2.5 | 2.5 | 98.4 |
|  | Other sources | 14 | 1.6 | 1.6 | 100.0 |
|  | Total | 893 | 100.0 | 100.0 |  |

CROSSTABS

/TABLES=media_exposure BY Health_service

/FORMAT=AVALUE TABLES

/STATISTICS=CHISQ PHI

/CELLS=COUNT ROW COLUMN

/COUNT ROUND CELL

/BARCHART.

**Crosstabs**

# Case Processing Summary

Cases

| Valid | | | Missing | | Total | |
| --- | --- | --- | --- | --- | --- | --- |
| N | | Percent | N | Percent | N | Percent |
| media_exposure * Health_service | 893 | 100.0% | 0 | 0.0% | 893 | 100.0% |

# media_exposure * Health_service Crosstabulation

Health_service

Public Health Services

| media_exposure | No Frequent Media Exposure | Count | 284 |
| --- | --- | --- | --- |
|  |  | % within media_exposure | 49.7% |
|  |  | % within Health_service | 64.5% |
|  | Frequent Media Exposure | Count | 156 |
|  |  | % within media_exposure | 48.4% |
|  |  | % within Health_service | 35.5% |
| Total | | Count | 440 |
|  |  | % within media_exposure | 49.3% |
|  |  | % within Health_service | 100.0% |

# media_exposure * Health_service Crosstabulation

Health_service

|  | | | Private Clinics\Pharma cies\Private Health Services | Chemists |
| --- | --- | --- | --- | --- |
| media_exposure | No Frequent Media Exposure | Count | 149 | 114 |
|  |  | % within media_exposure | 26.1% | 20.0% |
|  |  | % within Health_service | 62.3% | 64.0% |
|  | Frequent Media Exposure | Count | 90 | 64 |
|  |  | % within media_exposure | 28.0% | 19.9% |
|  |  | % within Health_service | 37.7% | 36.0% |
| Total | | Count | 239 | 178 |
|  |  | % within media_exposure | 26.8% | 19.9% |
|  |  | % within Health_service | 100.0% | 100.0% |

# media_exposure * Health_service Crosstabulation

Health_service

|  | | | Traditional Medicine |
| --- | --- | --- | --- |
| media_exposure | No Frequent Media Exposure | Count | 15 |
|  |  | % within media_exposure | 2.6% |
|  |  | % within Health_service | 68.2% |
|  | Frequent Media Exposure | Count | 7 |
|  |  | % within media_exposure | 2.2% |
|  |  | % within Health_service | 31.8% |
| Total | | Count | 22 |
|  |  | % within media_exposure | 2.5% |
|  |  | % within Health_service | 100.0% |

# media_exposure * Health_service Crosstabulation

| Health_service | | | | Total |
| --- | --- | --- | --- | --- |
|  | | | Other sources |  |
| media_exposure | No Frequent Media Exposure | Count | 9 | 571 |
|  |  | % within media_exposure | 1.6% | 100.0% |
|  |  | % within Health_service | 64.3% | 63.9% |
|  | Frequent Media Exposure | Count | 5 | 322 |
|  |  | % within media_exposure | 1.6% | 100.0% |
|  |  | % within Health_service | 35.7% | 36.1% |
| Total | | Count | 14 | 893 |
|  |  | % within media_exposure | 1.6% | 100.0% |
|  |  | % within Health_service | 100.0% | 100.0% |

**Chi-Square Tests**

| Value | | df | Asymptotic Significance (2- sided) |
| --- | --- | --- | --- |
| Pearson Chi-Square | .508a | 4 | .973 |
| Likelihood Ratio | .509 | 4 | .973 |
| Linear-by-Linear Association | .007 | 1 | .935 |
| N of Valid Cases | 893 |  |  |

a. 0 cells (0.0%) have expected count less than 5. The minimum expected count is 5.05.

# Symmetric Measures

| Value | | | Approximate Significance |
| --- | --- | --- | --- |
| Nominal by Nominal | Phi | .024 | .973 |
|  | Cramer's V | .024 | .973 |
| N of Valid Cases | | 893 |  |

**Bar Chart**

**Health_service**

Public Health Services

Private Clinics\Pharmacies\Private Health Services

Chemists Traditional Medicine Other sources

300

200

**Count**

100

0

No Frequent Media Exposure

Frequent Media Exposure

**media_exposure**

NOMREG Health_service (BASE=LAST ORDER=ASCENDING) BY media_exposure

/CRITERIA CIN(95) DELTA(0) MXITER(100) MXSTEP(5) CHKSEP(20) LCONVERGE(0) PCONVERGE(0.00 0001)

SINGULAR(0.00000001)

/MODEL

/STEPWISE=PIN(.05) POUT(0.1) MINEFFECT(0) RULE(SINGLE) ENTRYMETHOD(LR) REMOVALMETHOD(LR

)

/INTERCEPT=INCLUDE

/PRINT=PARAMETER SUMMARY LRT CPS STEP MFI.

**Nominal Regression**

**Case Processing Summary**

| N | | | Marginal Percentage |
| --- | --- | --- | --- |
| Health_service | Public Health Services | 440 | 49.3% |
|  | Private Clinics\Pharmacies\Private Health Services | 239 | 26.8% |
|  | Chemists | 178 | 19.9% |
|  | Traditional Medicine | 22 | 2.5% |
|  | Other sources | 14 | 1.6% |
| media_exposure | No Frequent Media Exposure | 571 | 63.9% |
|  | Frequent Media Exposure | 322 | 36.1% |
| Valid | | 893 | 100.0% |
| Missing | | 0 |  |
| Total | | 893 |  |
| Subpopulation | | 2 |  |

**Model Fitting Information**

| Model Fitting Criteria  -2 Log  Model Likelihood | | Likelihood Ratio Tests | | |
| --- | --- | --- | --- | --- |
|  |  | Chi-Square | df | Sig. |
| Intercept Only | 40.743 |  |  |  |
| Final | 40.234 | .509 | 4 | .973 |

**Pseudo R-Square**

| Cox and Snell | .001 |
| --- | --- |
| Nagelkerke | .001 |
| McFadden | .000 |

**Likelihood Ratio Tests**

| Model Fitting Criteria  -2 Log Likelihood of  Effect Reduced Model | | Likelihood Ratio Tests | | |
| --- | --- | --- | --- | --- |
|  |  | Chi-Square | df | Sig. |
| Intercept | 40.234a | .000 | 0 | . |
| media_exposure | 40.743 | .509 | 4 | .973 |

The chi-square statistic is the difference in -2 log-likelihoods between the final model and a reduced model. The reduced model is formed by omitting an effect from the final model. The null hypothesis is that all parameters of that effect are 0.

a. This reduced model is equivalent to the final model because omitting the effect does not increase the degrees of freedom.

# Parameter Estimates

| Health_servicea B | | | Std. Error | Wald | df |
| --- | --- | --- | --- | --- | --- |
| Public Health Services | Intercept | 3.440 | .454 | 57.344 | 1 |
|  | [media_exposure=.00] | .011 | .567 | .000 | 1 |
|  | [media_exposure=1.00] | 0b | . | . | 0 |
| Private Clinics\Pharmacies\Private Health Services | Intercept | 2.890 | .459 | 39.573 | 1 |
|  | [media_exposure=.00] | -.084 | .574 | .021 | 1 |
|  | [media_exposure=1.00] | 0b | . | . | 0 |
| Chemists | Intercept | 2.549 | .464 | 30.143 | 1 |
|  | [media_exposure=.00] | -.010 | .579 | .000 | 1 |
|  | [media_exposure=1.00] | 0b | . | . | 0 |
| Traditional Medicine | Intercept | .336 | .586 | .330 | 1 |
|  | [media_exposure=.00] | .174 | .722 | .058 | 1 |
|  | [media_exposure=1.00] | 0b | . | . | 0 |

| **Parameter Estimates** | | | | |
| --- | --- | --- | --- | --- |
|  | |  |  | 95%  Confidence ... |
| Health_servicea |  | Sig. | Exp(B) | Lower Bound |
| Public Health Services | Intercept | .000 |  |  |
|  | [media_exposure=.00] | .984 | 1.011 | .333 |
|  | [media_exposure=1.00] | . | . | . |
| Private | Intercept | .000 |  |  |
| Clinics\Pharmacies\Private  Health Services | [media_exposure=.00] | .884 | .920 | .299 |
|  | [media_exposure=1.00] | . | . | . |
| Chemists | Intercept | .000 |  |  |
|  | [media_exposure=.00] | .986 | .990 | .318 |
|  | [media_exposure=1.00] | . | . | . |
| Traditional Medicine | Intercept | .566 |  |  |
|  | [media_exposure=.00] | .809 | 1.190 | .289 |
|  | [media_exposure=1.00] | . | . | . |

Health_servicea

# Parameter Estimates

95% Confidence Interval for Exp...

Upper Bound

| Public Health Services | Intercept |  |
| --- | --- | --- |
|  | [media_exposure=.00] | 3.071 |
|  | [media_exposure=1.00] | . |
| Private Clinics\Pharmacies\Private Health Services | Intercept |  |
|  | [media_exposure=.00] | 2.830 |
|  | [media_exposure=1.00] | . |
| Chemists | Intercept |  |
|  | [media_exposure=.00] | 3.080 |
|  | [media_exposure=1.00] | . |
| Traditional Medicine | Intercept |  |
|  | [media_exposure=.00] | 4.897 |
|  | [media_exposure=1.00] | . |

1. The reference category is: Other sources.
2. This parameter is set to zero because it is redundant.
