# Supplementary material for: Socioeconomic and educational influences on malaria prevention and treatment behaviours in rural Nigeria
Source: BMC Public Health. 2025 Sep 24;25:3079. doi: 10.1186/s12889-025-24326-3 (PMC12462100; doi:10.1186/s12889-025-24326-3)
Supplement: Supplementary file 3 — Supplementary Material 3. [file 12889_2025_24326_MOESM3_ESM.docx]

USE ALL.

COMPUTE filter_$=(v025 = 2 AND NOT MISSING(Education) AND NOT MISSING(Prevention_awarene ss) AND

NOT MISSING(Use_of_prevention)).

VARIABLE LABELS filter_$ 'v025 = 2 AND NOT MISSING(Education) AND NOT '+

'MISSING(Prevention_awareness) AND NOT MISSING(Use_of_prevention) (FILTER)'.

VALUE LABELS filter_$ 0 'Not Selected' 1 'Selected'. FORMATS filter_$ (f1.0).

FILTER BY filter_$. EXECUTE.

FREQUENCIES VARIABLES=Education Prevention_awareness Use_of_prevention

/ORDER=ANALYSIS.

**Frequencies**

# Statistics

| Education | | | Prevention_aw areness | Use_of_prevent ion |
| --- | --- | --- | --- | --- |
| N | Valid | 9546 | 9546 | 9546 |
|  | Missing | 0 | 0 | 0 |

**Frequency Table**

**Education**

| Frequency | | | Percent | Valid Percent | Cumulative Percent |
| --- | --- | --- | --- | --- | --- |
| Valid | No Education | 3939 | 41.3 | 41.3 | 41.3 |
|  | Primary | 1409 | 14.8 | 14.8 | 56.0 |
|  | Secondary | 3352 | 35.1 | 35.1 | 91.1 |
|  | Tertiary | 846 | 8.9 | 8.9 | 100.0 |
|  | Total | 9546 | 100.0 | 100.0 |  |

**Prevention_awareness**

| Frequency | | | Percent | Valid Percent | Cumulative Percent |
| --- | --- | --- | --- | --- | --- |
| Valid | Not Aware | 3293 | 34.5 | 34.5 | 34.5 |
|  | Aware | 6253 | 65.5 | 65.5 | 100.0 |
|  | Total | 9546 | 100.0 | 100.0 |  |

**Use_of_prevention**

| Frequency | | | Percent | Valid Percent | Cumulative Percent |
| --- | --- | --- | --- | --- | --- |
| Valid | Does not use prevention method | 5543 | 58.1 | 58.1 | 58.1 |
|  | Uses prevention method | 4003 | 41.9 | 41.9 | 100.0 |
|  | Total | 9546 | 100.0 | 100.0 |  |

CROSSTABS

/TABLES=Education BY Prevention_awareness

/FORMAT=AVALUE TABLES

/STATISTICS=CHISQ

/CELLS=COUNT ROW

/COUNT ROUND CELL

/BARCHART.

**Crosstabs**

# Case Processing Summary

Cases

| Valid | | | Missing | | Total | |
| --- | --- | --- | --- | --- | --- | --- |
| N | | Percent | N | Percent | N | Percent |
| Education * Prevention_awareness | 9546 | 100.0% | 0 | 0.0% | 9546 | 100.0% |

# Education * Prevention_awareness Crosstabulation

| Prevention_awareness | | | | | Total |
| --- | --- | --- | --- | --- | --- |
| Not Aware | | | | Aware |  |
| Education | No Education | Count | 1485 | 2454 | 3939 |
|  |  | % within Education | 37.7% | 62.3% | 100.0% |
|  | Primary | Count | 529 | 880 | 1409 |
|  |  | % within Education | 37.5% | 62.5% | 100.0% |
|  | Secondary | Count | 1099 | 2253 | 3352 |
|  |  | % within Education | 32.8% | 67.2% | 100.0% |
|  | Tertiary | Count | 180 | 666 | 846 |
|  |  | % within Education | 21.3% | 78.7% | 100.0% |
| Total | | Count | 3293 | 6253 | 9546 |
|  |  | % within Education | 34.5% | 65.5% | 100.0% |

**Chi-Square Tests**

| Value | | df | Asymptotic Significance (2- sided) |
| --- | --- | --- | --- |
| Pearson Chi-Square | 93.451a | 3 | .000 |
| Likelihood Ratio | 98.636 | 3 | .000 |
| Linear-by-Linear Association | 69.560 | 1 | .000 |
| N of Valid Cases | 9546 |  |  |

a. 0 cells (0.0%) have expected count less than 5. The minimum expected count is 291.84.

2,500

2,000

1,500

**Count**

1,000

500

**Bar Chart**

0

**Prevention_awareness**

Not Aware Aware

No Education

Primary

Secondary

Tertiary

**Education**

CROSSTABS

/TABLES=Education BY Use_of_prevention

/FORMAT=AVALUE TABLES

/STATISTICS=CHISQ

/CELLS=COUNT ROW

/COUNT ROUND CELL

/BARCHART.

**Crosstabs**

# Case Processing Summary

Cases

| Valid | | | Missing | | Total | |
| --- | --- | --- | --- | --- | --- | --- |
| N | | Percent | N | Percent | N | Percent |
| Education * Use_of_prevention | 9546 | 100.0% | 0 | 0.0% | 9546 | 100.0% |

# Education * Use_of_prevention Crosstabulation

| Use_of_prevention | | | | | Total |
| --- | --- | --- | --- | --- | --- |
| Does not use prevention method | | | | Uses prevention method |  |
| Education | No Education | Count | 1931 | 2008 | 3939 |
|  |  | % within Education | 49.0% | 51.0% | 100.0% |
|  | Primary | Count | 751 | 658 | 1409 |
|  |  | % within Education | 53.3% | 46.7% | 100.0% |
|  | Secondary | Count | 2242 | 1110 | 3352 |
|  |  | % within Education | 66.9% | 33.1% | 100.0% |
|  | Tertiary | Count | 619 | 227 | 846 |
|  |  | % within Education | 73.2% | 26.8% | 100.0% |
| Total | | Count | 5543 | 4003 | 9546 |
|  |  | % within Education | 58.1% | 41.9% | 100.0% |

**Chi-Square Tests**

| Value | | df | Asymptotic Significance (2- sided) |
| --- | --- | --- | --- |
| Pearson Chi-Square | 331.761a | 3 | .000 |
| Likelihood Ratio | 336.794 | 3 | .000 |
| Linear-by-Linear Association | 320.554 | 1 | .000 |
| N of Valid Cases | 9546 |  |  |

a. 0 cells (0.0%) have expected count less than 5. The minimum expected count is 354.76.

2,500

2,000

1,500

**Count**

1,000

500

**Bar Chart**

0

**Use_of_prevention**

Does not use prevention metho Uses prevention method

No Education

Primary

Secondary

Tertiary

**Education**

LOGISTIC REGRESSION VARIABLES Prevention_awareness

/METHOD=ENTER Education

/CONTRAST (Education)=Indicator

/CRITERIA=PIN(.05) POUT(.10) ITERATE(20) CUT(.5).

**Logistic Regression**

# Case Processing Summary

| Unweighted Casesa N | | | Percent |
| --- | --- | --- | --- |
| Selected Cases | Included in Analysis | 9546 | 100.0 |
|  | Missing Cases | 0 | .0 |
|  | Total | 9546 | 100.0 |
| Unselected Cases | | 0 | .0 |
| Total | | 9546 | 100.0 |

1. If weight is in effect, see classification table for the total number of cases.

# Dependent Variable Encoding

Original Value Internal Value

| Not Aware | 0 |
| --- | --- |
| Aware | 1 |

# Categorical Variables Codings

| Frequency | | | Parameter coding | | |
| --- | --- | --- | --- | --- | --- |
|  |  |  | (1) | (2) | (3) |
| Education | No Education | 3939 | 1.000 | .000 | .000 |
|  | Primary | 1409 | .000 | 1.000 | .000 |
|  | Secondary | 3352 | .000 | .000 | 1.000 |
|  | Tertiary | 846 | .000 | .000 | .000 |

**Block 0: Beginning Block**

# Classification Tablea,b

Predicted

| Prevention_awareness | | | | | Percentage Correct |
| --- | --- | --- | --- | --- | --- |
| Observed Not Aware | | | | Aware |  |
| Step 0 | Prevention_awareness | Not Aware | 0 | 3293 | .0 |
|  |  | Aware | 0 | 6253 | 100.0 |
|  | Overall Percentage | |  |  | 65.5 |

1. Constant is included in the model.
2. The cut value is .500

# Variables in the Equation

| B | | S.E. | Wald | df | Sig. | Exp(B) |
| --- | --- | --- | --- | --- | --- | --- |
| Step 0 Constant | .641 | .022 | 887.013 | 1 | .000 | 1.899 |

**Variables not in the Equation**

| Score | | | | df | Sig. |
| --- | --- | --- | --- | --- | --- |
| Step 0 | Variables | Education | 93.451 | 3 | .000 |
|  |  | Education(1) | 30.463 | 1 | .000 |
|  |  | Education(2) | 6.797 | 1 | .009 |
|  |  | Education(3) | 6.683 | 1 | .010 |
|  | Overall Statistics | | 93.451 | 3 | .000 |

**Block 1: Method = Enter**

# Omnibus Tests of Model Coefficients

| Chi-square | | | df | Sig. |
| --- | --- | --- | --- | --- |
| Step 1 | Step | 98.636 | 3 | .000 |
|  | Block | 98.636 | 3 | .000 |
|  | Model | 98.636 | 3 | .000 |

**Model Summary**

| -2 Log  Step likelihood | | Cox & Snell R Square | Nagelkerke R Square |
| --- | --- | --- | --- |
| 1 | 12201.796a | .010 | .014 |

1. Estimation terminated at iteration number 4 because parameter estimates changed by less than .001.

# Classification Tablea

Predicted

| Prevention_awareness | | | | | Percentage Correct |
| --- | --- | --- | --- | --- | --- |
| Observed Not Aware | | | | Aware |  |
| Step 1 | Prevention_awareness | Not Aware | 0 | 3293 | .0 |
|  |  | Aware | 0 | 6253 | 100.0 |
|  | Overall Percentage | |  |  | 65.5 |

1. The cut value is .500

# Variables in the Equation

| B | | | S.E. | Wald | df | Sig. | Exp(B) |
| --- | --- | --- | --- | --- | --- | --- | --- |
| Step 1a | Education |  |  | 90.829 | 3 | .000 |  |
|  | Education(1) | -.806 | .090 | 79.834 | 1 | .000 | .447 |
|  | Education(2) | -.799 | .100 | 63.373 | 1 | .000 | .450 |
|  | Education(3) | -.590 | .092 | 41.453 | 1 | .000 | .554 |
|  | Constant | 1.308 | .084 | 242.556 | 1 | .000 | 3.700 |

1. Variable(s) entered on step 1: Education.

LOGISTIC REGRESSION VARIABLES Use_of_prevention

/METHOD=ENTER Education

/CONTRAST (Education)=Indicator

/CRITERIA=PIN(.05) POUT(.10) ITERATE(20) CUT(.5).

**Logistic Regression**

# Case Processing Summary

| Unweighted Casesa N | | | Percent |
| --- | --- | --- | --- |
| Selected Cases | Included in Analysis | 9546 | 100.0 |
|  | Missing Cases | 0 | .0 |
|  | Total | 9546 | 100.0 |
| Unselected Cases | | 0 | .0 |
| Total | | 9546 | 100.0 |

1. If weight is in effect, see classification table for the total number of cases.

# Dependent Variable Encoding

Original Value Internal Value

| Does not use prevention method | 0 |
| --- | --- |
| Uses prevention method | 1 |

# Categorical Variables Codings

| Frequency | | | Parameter coding | | |
| --- | --- | --- | --- | --- | --- |
|  |  |  | (1) | (2) | (3) |
| Education | No Education | 3939 | 1.000 | .000 | .000 |
|  | Primary | 1409 | .000 | 1.000 | .000 |
|  | Secondary | 3352 | .000 | .000 | 1.000 |
|  | Tertiary | 846 | .000 | .000 | .000 |

**Block 0: Beginning Block**

# Classification Tablea,b

Predicted

Use_of_prevention

Step 0

Observed

Use_of_prevention

Does not use prevention method

Uses prevention method

Does not use prevention method

Uses prevention method

Overall Percentage

0

4003

0

5543

# Classification Tablea,b

Predicted

Observed

Percentage Correct

| Step 0 | Use_of_prevention | Does not use prevention method | 100.0 |
| --- | --- | --- | --- |
|  |  | Uses prevention method | .0 |
|  | Overall Percentage | | 58.1 |

1. Constant is included in the model.
2. The cut value is .500

# Variables in the Equation

| B | | S.E. | Wald | df | Sig. | Exp(B) |
| --- | --- | --- | --- | --- | --- | --- |
| Step 0 Constant | -.325 | .021 | 246.257 | 1 | .000 | .722 |

**Variables not in the Equation**

| Score | | | | df | Sig. |
| --- | --- | --- | --- | --- | --- |
| Step 0 | Variables | Education | 331.761 | 3 | .000 |
|  |  | Education(1) | 225.254 | 1 | .000 |
|  |  | Education(2) | 15.420 | 1 | .000 |
|  |  | Education(3) | 165.017 | 1 | .000 |
|  | Overall Statistics | | 331.761 | 3 | .000 |

**Block 1: Method = Enter**

# Omnibus Tests of Model Coefficients

| Chi-square | | | df | Sig. |
| --- | --- | --- | --- | --- |
| Step 1 | Step | 336.794 | 3 | .000 |
|  | Block | 336.794 | 3 | .000 |
|  | Model | 336.794 | 3 | .000 |

**Model Summary**

| -2 Log  Step likelihood | | Cox & Snell R Square | Nagelkerke R Square |
| --- | --- | --- | --- |
| 1 | 12647.244a | .035 | .047 |

1. Estimation terminated at iteration number 4 because parameter estimates changed by less than .001.

# Classification Tablea

Predicted

Use_of_prevention

Step 1

Observed

Use_of_prevention

Does not use prevention method

Uses prevention method

Does not use prevention method

Uses prevention method

Overall Percentage

2008

1995

1931

3612

# Classification Tablea

Predicted

Observed

Percentage Correct

| Step 1 | Use_of_prevention | Does not use prevention method | 65.2 |
| --- | --- | --- | --- |
|  |  | Uses prevention method | 50.2 |
|  | Overall Percentage | | 58.9 |

1. The cut value is .500

# Variables in the Equation

| B | | | S.E. | Wald | df | Sig. | Exp(B) |
| --- | --- | --- | --- | --- | --- | --- | --- |
| Step 1a | Education |  |  | 325.527 | 3 | .000 |  |
|  | Education(1) | 1.042 | .084 | 154.377 | 1 | .000 | 2.836 |
|  | Education(2) | .871 | .094 | 85.500 | 1 | .000 | 2.389 |
|  | Education(3) | .300 | .086 | 12.227 | 1 | .000 | 1.350 |
|  | Constant | -1.003 | .078 | 167.141 | 1 | .000 | .367 |

1. Variable(s) entered on step 1: Education.
